# A Road Map for 21st Century Genetic Restoration: Gene Pool Enrichment of the Black-Footed Ferret

**DOI:** 10.1093/jhered/esv041

**Published:** 2015-08-24

**Authors:** Samantha M. Wisely, Oliver A. Ryder, Rachel M. Santymire, John F. Engelhardt, Ben J. Novak

**Affiliations:** From the Department of Wildlife Ecology and Conservation, University of Florida, 110 Newins-Ziegler Hall, Gainesville, Florida, 32611USA (Wisely); San Diego Zoo Institute for Conservation Research, 15600 San Pasqual Valley Road, San Diego Zoo Global, Escondido, California, 92027USA (Ryder); Davee Center for Epidemiology and Endocrinology, 2001 North Clark Street, Lincoln Park Zoo, Chicago, Illinois, 60614USA (Santymire); Department of Anatomy and Cell Biology, 51 Newton Road, University of Iowa, Iowa City, Iowa, 52242USA (Engelhardt); and Revive & Restore, The Long Now Foundation, 2 Marina Boulevard Building A, San Francisco, California, 94123USA (Novak).

**Keywords:** conservation cloning, endangered species management, *Mustela nigripes*, somatic cell nuclear transfer

## Abstract

Interspecies somatic cell nuclear transfer (iSCNT) could benefit recovery programs of critically endangered species but must be weighed with the risks of failure. To weigh the risks and benefits, a decision-making process that evaluates progress is needed. Experiments that evaluate the efficiency and efficacy of blastocyst, fetal, and post-parturition development are necessary to determine the success or failure or species-specific iSCNT programs. Here, we use the black-footed ferret (*Mustela nigripes*) as a case study for evaluating this emerging biomedical technology as a tool for genetic restoration. The black-footed ferret has depleted genetic variation yet genome resource banks contain genetic material of individuals not currently represented in the extant lineage. Thus, genetic restoration of the species is in theory possible and could help reduce the persistent erosion of genetic diversity from drift. Extensive genetic, genomic, and reproductive science tools have previously been developed in black-footed ferrets and would aid in the process of developing an iSCNT protocol for this species. Nonetheless, developing reproductive cloning will require years of experiments and a coordinated effort among recovery partners. The information gained from a well-planned research effort with the goal of genetic restoration via reproductive cloning could establish a 21st century model for evaluating and implementing conservation breeding that would be applicable to other genetically impoverished species.

Genetic restoration has the potential to save highly endangered species from the grips of an extinction vortex by increasing mean population fitness to a level that stabilizes population growth (Ingvarsson 2001). The concept of genetic restoration is the result of 2 complementary ideas: that the deleterious effects of inbreeding can contribute to population extinction (Gilpin and Soulé 1986) and that novel genetic variation from immigrants can alleviate those deleterious effects (Spielman and Frankham 1992). Empirical evidence from plants, insects, and vertebrates continues to accumulate and suggests that genetic restoration via the migration of just a few individuals into a small, genetically depauperate population increases average population fitness (reviewed by Tallmon et al. 2004). Increasingly, genetic restoration is being used as a management tool for imperiled populations. The Florida panther (*Puma concolor coryi*) provides an example of the power of genetic restoration as a management tool for *in situ* conservation. A population on the verge of extinction, Florida panthers showed classic symptoms of inbreeding depression: cryptorchidism, heart defects, and declining reproduction (Roelke et al. 1993; Barone et al. 1994; Culver et al. 2000). The introduction of 8 Texas panther females (*Puma concolor stanleyana*) into the remaining isolated population of 20–25 Florida panthers in 1995 led to positive population response (Hostetler et al. 2013) and alleviation of physiological abnormalities associated with inbreeding depression (Johnson et al. 2010). Today, there are between 100 and 160 animals, and the population maintains a positive growth rate (Hostetler et al. 2013). The use of assisted and unassisted translocations for the purpose of genetic restoration is now a common management tool for endangered species (Whiteley et al. 2014).

In parallel to the management of *in situ* populations, conservation breeding of *ex situ* threatened and endangered wildlife has also augmented gene pool diversity of captive populations to avoid inbreeding [e.g. pygmy rabbit (*Brachylagus idahoensis*) Elias et al. 2013; Puerto Rican crested toad (*Peltophryne lemur*) Beauclerc et al. 2010; Spanish killifish (*Aphanius baeticus*) Schönhuth et al. 2003]. Genetic restoration can be achieved by bringing new individuals into captivity or via assisted breeding. Assisted breeding and cryotechnology can be essential to genetic restoration for species that are difficult to breed in captivity, to maintain genetically valuable lineages after animals have died, or to incorporate new genetic founders from cryopreserved, curated genomic resources (Wildt et al. 2003). Once the purview of domestic breeders, assisted breeding technologies have aided in the captive breeding of many species including the giant panda (*Ailuropoda melanoleuca*; Wildt et al. 2003), endangered felids (e.g. Swanson 2012), parrots (Lierz et al. 2013), as well as other nonmammal species (Comizzoli et al. 2009, 2012).

One technology which has emerged as a useful tool for biomedical research is somatic cell nuclear transfer (SCNT; Campbell et al. 1996) which enables the production of individuals with the same nuclear DNA content (reproductive cloning, Figure 1) from cells other than germ cells (e.g. skin cells). SCNT is used regularly to produce transgenic animals that model human diseases. The value of this technology to the conservation goals of endangered species was quickly recognized as a possible tool for increasing genetic variability in species that had few founders or very low genetic diversity (Ryder and Benirschke 1997; Ryder 2002; Holt et al. 2004). The difficulties of this technology became apparent as different research groups had low levels of success with interspecies SCNT (Table 1). The nuances of species-specific embryology, reproductive biology, chromosome architecture, maternal influences, and cytology make each cloning attempt a species-specific endeavor. These technical challenges and their associated costs are a reason that the conservation community has been slow to embrace reproductive cloning for genetic restoration. Nonetheless, for highly endangered species that have a small effective population size and rely on captive breeding for their existence, the potential benefit of augmenting these small, closed gene pools with unique genetic contributions that would otherwise be unavailable is too great to ignore.

**Figure 1.**
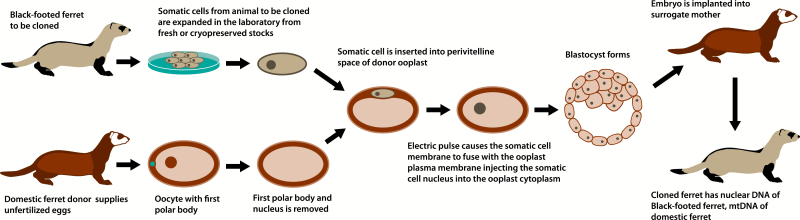
A conceptual model for interspecies somatic cell nuclear transfer in reproductive cloning of black-footed ferrets (*Mustela nigripes*). (**a**) The first polar body and nucleus are removed from the domestic ferret oocyte. (**b**) Somatic cells from black-footed ferrets are expanded in the laboratory from fresh or cryopreserved stocks. (**c**) The somatic cell of the black-footed ferret is inserted and then fused with the domestic ferret oocyte. Oocytes have the ability to reprogram exogenous and endogenous genetic material to allow normal embryonic development. Harnessing that ability to program the nucleus to begin embryonic development is an essential step in SCNT and reproductive cloning (Gurdon and Melton 2008).

**Table 1.** Examples of reproductive cloning using iSCNT in endangered wildlife and the outcome of the effort. Inefficiencies in establishing blastocysts from oocytes, establishing pregnancies, and producing live, viable births dominate the literature on iSCNT

**Species**	**Source of genetic material**	**Source of oocytes**	**Outcome**	**Reference**
Gaur (*Bos gaurus*) 2*n* = 58	Dermal fibroblasts from postmortem male	Domestic cattle (*Bos taurus*) 2*n* = 60	81 blastocysts from 692 oocytes, 8 of 32 pregnancies established, 1 live birth resulted that died 2 days post parturition	Lanza et al. 2000
African wild cat (*Felis silvestris lybica*) 2*n* = 38	Crypreserved fibroblasts	Domestic cat (*Felis silvestris catus*) 2*n* = 38	No. blastocysts from 2432 oocytes not reported, 12 of 26 established pregnancies, 17 kittens were born, 2 survived	Gómez et al. 2004
Gray wolf (*Canis lupus*) 2*n* = 78	Dermal fibroblast from postmortem animal	Domestic dog (*Canis l. familiaris*) 2*n* = 78	No. blastocysts derived from oocytes not reported, 4 of 17 pregnancies established, 3 of 6 clones survived past parturition	Kim et al. 2007 Oh et al. 2008
Sand cat (*Felis margarita*) 2*n* = 38	Cryopreserved fibroblast	Domestic cat (*Felis silvestris*) 2*n* = 38	83 blastocysts derived from 1282 oocytes, 14 of 45 pregnancies established, 5 of 14 clones survived past parturition. All died by 60 days post parturition	Gómez et al. 2008
Pyrenean ibex (*Capra pyrenaica*) 2*n* = 60	Cryopreserved fibroblasts	Domestic goat (*Capra aegagrus hircus*) 2*n* = 60	No. blastocysts derived from oocytes not reported, 1 of 44 pregancies established, 1 of 5 survived past parturition but died shortly thereafter.	Folch et al. 2009
Esfahan mouflon (*Ovis orientalis isphahanica*) 2*n* = 54	Cryopreserved fibroblasts	Domestic sheep (*Ovis aries*) 2*n* = 54	96 blastocysts from 667 oocytes, 2 of 5 pregnancies established, 2 live births that died post parturition.	Hajian et al. 2011
Coyote (*Canis latrans*) 2*n* = 78	Neonatal fibroblasts	Domestic dog (*Canis familiaris*) 2*n* = 78	No. blastocysts derived from oocytes not reported. 6 of 22 pregnancies established, 5 live births to 3 mothers.	Hwang 2013

Nearly all of the recent reviews of genetic restoration by reproductive cloning have been published in reproductive biology or cloning journals (Holt et al. 2004; Piña-Aguilar et al. 2009; Loi et al. 2011), and thus, many conservation geneticists have not recently considered this technology for highly imperiled species. In this paper, we seek to clarify the procedures necessary to achieve genetic restoration via reproductive cloning. We address the unique benefits that this procedure would bring to conservation objectives, the limits of this technology, and the technical difficulties that currently prevent the method from being widely used. We then use the black-footed ferret as a case study of a species that could benefit from reproductive cloning. We outline the benefits and challenges of reproductive cloning in black-footed ferrets and then provide a roadmap of steps that would be necessary to produce a proof of concept based on the current state of understanding of the reproductive biology and genetics of this species. Ultimately, we hope that this document generates dialog among conservation practitioners who seek new ways to increase the effective population size of critically endangered species.

## Cloning Using SCNT

Reproductive cloning via SCNT allows for the genetic duplication of an individual. What made SCNT revolutionary was that it eliminated the need for the desired genetic material to come from a germ cell; genomes of interest could, in principle, come from any somatic cell. In practice, these cells are often fibroblasts in cell culture. A donor oocyte, whose nucleus has been removed, becomes the cellular vessel that holds the diploid genome from a different organism. Depending on the method of fusion used, the donor ooplast may retain the cytoplasmic architecture of its donor including mitochondrial DNA, RNA, and other organelles in the cytoplasm or a mix of cytoplasmic material from the egg and the nucleus donors (Figure 1). Donor oocytes can be harvested from recently deceased animals, from animals whose ovaries are surgically removed, or by aspiration of oocytes without ovariectomy. The oocytes infused with foreign nuclear material develop into reconstructed embryos which are then cultured and allowed to develop into blastocysts *in vitro* and then implanted surgically or by nonsurgical methods into surrogate females that carry the developing clone through to parturition. The resulting animal is considered a clone of the donor of the somatic cell.

The process of reproductive cloning is inefficient. Many enucleated oocytes must be merged with many nuclei to produce viable clonal embryos, and not all embryos develop to term (Table 1). As conservation breeders began to consider reproductive cloning to increase or maintain gene pool diversity in endangered species, it became clear that harvesting ovaries from endangered species would not be practical or desirable. Thus, cloning for conservation has embraced interspecies somatic cell nuclear transfer (iSCNT) for assisted breeding, whereby the donated oocytes come from nonendangered species which are often closely related domestic species (Table 1 and references therein).

## The Benefits and Challenges of iSCNT

Genetic restoration has been shown to provide phenotypic and demographic benefits to multiple endangered species. Yet, for many endangered species (e.g. black-footed ferret, California Condor, Prezwalski’s horse) the opportunity no longer exists to add genetically unique immigrants from the wild, because wild populations are extinct. Curated, frozen repositories of somatic and germ cells (biological resource banks) have been created for the purpose of both assisted breeding and reproductive cloning. These collections provide unique genetic resources to these critically endangered species with the goal of increasing the effective population size of the species. For example, cryopreservation of sperm has successfully been used to genetically augment many captive breeding programs (e.g. black-footed ferret (Howard and Wildt 2009), giant panda (Wildt et al. 1993), cheetah (Howard et al. 1992), and Eld’s deer (Monfort et al. 1993)), but biological resource banks have had less success preserving other germ tissues such as frozen embryos or eggs (Saragusty et al. 2011). In some cases, collecting germ cells (e.g. sperm, eggs, or their ontological precursors) from genetically valuable animals that are unrepresented or underrepresented in a captive breeding pedigree is not possible. Situations may arise when the animal is too valuable to submit to invasive collection procedures or germ cells have not been preserved as part of the biological resource collection. In these cases, somatic cells (e.g. skin or other epithelial cells) collected from live animals or retrieved from biological resource banks may be the only source for genetic restoration, and iSCNT would provide a way to integrate genetically unique contributions in to the gene pool. More than 20 zoos and aquariums participate in biological resource banks (e.g. http://www.frozenark.org/), and regional biological resource banks have been created for southern African wildlife (Bartels and Kotze 2006) and critically endangered wildlife species (e.g. Iberian lynx, Leon-Quinto et al. 2009).

For intraspecies SCNT, the source of somatic cells has been diverse, including tissue frozen without a cryoprotectant (Loi et al. 2008; Wakayama et al. 2008; Hoshino et al. 2009; Cetinkaya et al. 2014), tissue from permafrost animals (Kato et al. 2009), cells collected postmortem (Loi et al. 2001; Oh et al. 2008), and somatic cells obtained from semen (Nel-Themaat et al. 2007; Nel-Themaat et al. 2008; Liu et al. 2010). Nonetheless, quality of collected cells contributes substantially to efficiency of blastocyst development which is already low in iSCNT-produced embryos (Table 1). Therefore, collecting genomic material from cells that have been frozen but not cryopreserved reduces the already low success rate. As SCNT technologies improve, it can be possible to consider noncryopreserved sources of genetic material for inclusion in the gene pool and the benefits that they might offer genetically depauperate species.

Despite the benefits that iSCNT holds for endangered species conservation, examples of successful use of iSCNT are rare (Table 1) because of the low number of viable births that are produced from this procedure. This inefficiency is largely the result of an insufficient number of oocytes for iSCNT trials or the incompatibility of nuclear material from one species with the cytoplasmic material of another species which inhibits blastocyst formation. One such incompatibility is the inappropriate genetic reprogramming by the donor oocyte of the donor nucleus. Genetic reprogramming is a critical step in early embryonic development that relies on epigenetic interactions of maternal cytoplasmic RNA and proteins to remove and remodel the existing methylation pattern on the inherited, or in the case of reproductive cloning, injected chromatin. When there is appropriate reprogramming by the oocyte and activation of the donated genome, cell differentiation and development follows a predictable path to produce a normal embryo. When cytoplasmic material and genetic material are from different species, inappropriate gene expression and therefore improper cell differentiation and physiological development can occur during the growth of the embryo and fetus, and even after parturition (Gurdon and Melton 2008). Recent research has shown that inappropriate genetic reprogramming can be mitigated with chemical treatment of donor chromatin to change methylation patterns involved in gene expression (Loi et al. 2011). This technique has been used with success in iSCNT for *in vitro* development of blastocysts of the endangered black-footed cat (*Felis nigripes*, Gómez et al. 2012).

While epigenetic effects can inhibit normal development, so can incompatibilities between the oocyte mtDNA genome and the donated nuclear genome. ATP synthesis and mitochondrial replication require coordinated gene expression of the mitochondrial and nuclear genes responsible for those functions. The incompatibility of the nuclear and mitochondrial genome can result in reduced mitochondrial efficiency, apoptosis, and abnormal development in iSCNT embryos (reviewed by Loi et al. 2011; Lagutina et al. 2013). To decrease the effect of this incompatibility, cytoplasm from the nucleus donor has been inserted along with the nucleus into the donor oocyte to create a heteroplasmic oocyte (cybrid) (Jiang et al. 2011). Cybrids appear to have fewer mitochondrial incompatibility issues and increased efficiency in blastocyst development. While it is clear that incompatibility increases as phylogenetic distance increases between mitochondrial and nuclear genome donors (Loi et al. 2011), further studies are needed to understand the potential benefit and the metabolic consequences of cybrids in iSCNT.

In addition to advancing species-specific iSCNT technology to the point where reconstructed embryos are produced, suitable embryo recipient species capable of carrying the embryo to term must be found. This vetting would ideally start with reciprocal embryo implant experiments between the oocyte and nucleus donor species without iSCNT embryos to determine compatibility without the confounding influence of iSCNT inefficiency. For the case of the bucardo (*Capra pyrenaica pyrenaica*), differences in gestation length prohibited the use of domestic goats as foster mothers, and F1 hybrids were ultimately used (Folch et al. 2009). Factors other than incompatibilities also influence iSCNT efficiency. Somatic cell type, cryopreservation history, and the maturation stage of the donor oocyte influence iSCNT success (reviewed by Thongphakdee et al. 2010).

**Figure 2.**
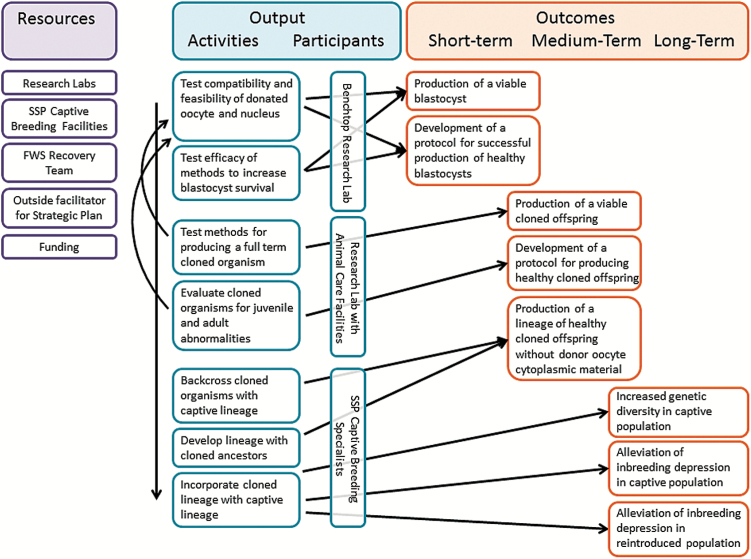
Logic framework for developing a research plan with the goal of genetic restoration of black-footed ferrets via reproductive cloning.

Despite the hurdles to increasing the efficiency and success of iSCNT, SCNT across a breadth of phylogenetic distances has become a major experimental pathway to better understand the developmental architecture of embryos that leads to pluripotency (cell differentiation) and totipotency (total embryonic differentiation) (Sun and Zhu 2014). Because nuclear reprogramming and resulting pluripotency is an essential component of stem cell research in biomedicine, continued investment and advancements in this area of research will occur (Gurdon and Melton 2008). While these advances will inevitably help advance iSCNT for conservation breeding, well-designed iSCNT experiments within an endangered species study system will be necessary given the unique embryology of each species, even if they are not successful in obtaining offspring. Designing experiments in a systematic and logical manner will be critical to understanding of the developmental and reproductive biology of the focal species in addition to the long-term goal of reproductive cloning for conservation (Figure 2).

## Black-Footed Ferrets as a Case Study for Using iSCNT in Endangered Species Recovery

Existing reproductive and developmental resources for black-footed ferrets greatly surpass what is available for the vast majority of currently endangered species. The species was used to pioneer assisted breeding in endangered species, and thus, many details are known about the reproductive biology of this species (Howard et al. 2003). SCNT has been developed in domestic ferrets (Li et al. 2006), and this cloning technology has been used to create powerful transgenic models for the study of human diseases (Olivier et al. 2012). These advancements set the stage to discuss the use of iSCNT to increase the genetic diversity in the gene pool of black-footed ferrets by introducing genetic variation from extinct lineages.

## Conservation Genetics of the Black-Footed Ferret

Black-footed ferrets are an excellent candidate for genetic restoration. They have been considered one of the most endangered mammals in North America, and, in fact, were twice presumed extinct in the recent past. Once widespread across the Great Plains and intermountain grasslands of the Rockies, habitat loss, persecution of their main prey item, the prairie dog (*Cynomys* spp.), and ultimately disease reduced the species to one population. A century of local population extirpation resulted in a 30–50% loss of genetic diversity in the species (Wisely et al. 2002). In 1981, epizootics of canine distemper virus (CDV) and sylvatic plague threatened this population with extinction. In response to this threat, in 1986, the US Fish and Wildlife Service and Wyoming Game and Fish Department made the historic decision to manage an endangered species by capturing all of the remaining wild individuals and bringing them into captivity to begin a conservation breeding program with ultimate goal of restoring the species to its former range. Of the 18 individuals captured at Meeteetse, Wyoming, only 7 individuals of unknown relatedness (3 males and 4 females) became the founding population of all black-footed ferrets alive today. These 7 individuals represented 70–85% of the genetic variation that was present in the Meeteetse population (Wisely et al. 2002). Thus, the genetic variation of those 7 founders and in contemporary descendant lineages is only a portion of the total genetic variation in the species (Wisely 2005).

From those 7 founders, >8700 ferrets have been produced in 27 years of captive breeding. The captive population has been actively managed to conserve the maximum amount of genetic diversity using mate pairing through consideration of mean kinship (Ballou and Lacy 1995). More than 25 years of intensively managed breeding has resulted in preserving 86% of the original gene diversity present in the founding population, with the majority of loss likely occurring in the early generations of the program (Garelle et al. 2013). The genetic diversity retained reflects the successful efforts of the program to remain within the goal set by the Black-Footed Ferret Recovery Plan (USFWS 2013b), but further loss is inevitable from genetic drift and represents a gradual erosion of genetic variability.

Although the captive population has stabilized and kit production has become efficient, major hurdles remain for *in situ* conservation which creates the need for a healthy and diverse *ex situ* population. Within the Great Plains of North America, the pervasive and expanding distribution of sylvatic plague limits the success of reintroduced populations, while lack of suitable habitat slows the pace of new reintroductions. Since 1991, approximately 4200 black-footed ferrets have been released into 21 sites in Canada, the United States, and northern Mexico. Currently, approximately 440 individuals are surviving and reproducing in 14 sites (USFWS 2013a). It should therefore be anticipated that black-footed ferret recovery requires a productive captive population for at least the next 50 years while habitat is restored. In order to achieve that goal, a healthy captive population able to withstand the inevitable erosion of genetic diversity and increase in inbreeding is required to contribute to recovery.

Despite ongoing success in the *ex situ* population, indications of inbreeding, inbreeding depression, and domestication are present. According to the Black-footed Ferret Species Survival Plan (Garelle et al. 2013), there has been a decrease in whelping rates in females and normal sperm in males (Wolf et al. 2000; Santymire et al. 2006, 2007, 2014a, 2014b) and changes in both male and female fecundity (Santymire et al. 2014a,b). In other carnivoran species, these traits have been linked to inbreeding depression, e.g., in lions, *Panthera leo*, (Munson et al. 1996; Wildt et al. 1987); cheetahs, *Acinonyx jubatus*, (Wildt et al. 1983; O’Brien et al. 1985); and Florida panthers (Roelke et al. 1993; Barone et al. 1994). In addition, losses of immune function have been observed (Kennedy-Stoskopf et al. 1997). It has also been anecdotally observed that litter size has increased in captive animals, which is a trait commonly associated with domestication (Trut 1999). The relative contribution of environmental vs. genetic causes of these changes in fitness remains unclear, and additional assessment is needed. Yet even without solid evidence that these phenotypic changes are a result of inbreeding depression, genetic augmentation of black-footed ferrets can be justified given the anticipated length of time the *ex situ* population will be needed, the paucity of genetic diversity in the founding individuals, and the ongoing effects of genetic drift.

## Genetic Augmentation of the Gene Pool in Black-Footed Ferrets

Since initiation of the captive breeding program, proactive management of genetic diversity has been a hallmark of the stewardship of this species. The black-footed ferret captive breeding program was one of the first to utilize a species survival plan that used science-based breeding strategies to maximize retention of genetic diversity through time (Wisely et al. 2003). To enhance those breeding strategies, the recovery program embraced reproductive technologies to slow the pace of genetic drift in the captive population (Howard et al. 2003). Over the past 20 years, scientists working with the Recovery Team developed artificial insemination to ensure that males who did not reproduce or were underrepresented in the gene pool could contribute, even after those animals had died. To date, approximately 140 black-footed ferrets have been produced via artificial insemination including 8 that were produced from semen that had been cryopreserved for 10–20 years. This form of assisted breeding helped to incorporate lost genes back into the gene pool, lengthen generation time, and slow the pace of genetic drift that occurred as the result of decades of captive breeding (Howard et al. forthcoming). Genome resource banking for this species began early in the recovery program, and its development and maintenance is ongoing (USFWS 2013b). Resources include cryopreserved sperm collected at different time points in the captive breeding program, from multiple, wild-born descendant populations (Santymire et al. 2004) and from cryopreserved fibroblast cell lines that were established from 2 black-footed ferrets from the Meeteetse population that are not represented in the existing lineage. Each of these resources represents genetic material that has the capacity to increase diversity in the extant captive breeding population.

While cryopreserved sperm has been used in the assisted breeding of multiple endangered species, the use of somatic cells to increase or maintain genetic diversity has not. Yet, the ongoing loss of genetic diversity via drift and the need for *ex situ* breeding for the foreseeable future make additional genetic restoration of this species via iSCNT worthy of consideration. This consideration is further bolstered by the accumulation of knowledge of the reproductive biology of this species and the convergence of reproductive, genomic, and developmental technologies for the black-footed ferret and a congener, the domestic ferret (*Mustela putorius furo*). Indeed, the black-footed ferret has previously been suggested as a candidate for conservation cloning (Piña-Aguilar et al. 2009). Potential resources available for genetic restoration include: 1) Novel conspecific genomes from the same population as the founders that can be added to the current gene pool. Forward thinking conservation practitioners have collected and cryopreserved biological material from black-footed ferrets not represented in the founder population; 2) Conspecific genomes from genetically valuable but deceased animals from the captive breeding population have been cryopreserved; 3) Genomic resources for ferrets are available and are being further developed. Whole genomes from 4 black-footed ferrets including the cell lines from unrepresented individuals have been sequenced (NCBI Bioproject PRJNA254451). The domestic ferret genome is currently available (Peng et al. 2014) and European ferret and Siberian polecat genomes will soon be published (Di Palma F, personal communication) such that comparative genomic studies of this clade can commence; 4) A congener cloning model has been developed. Domestic ferrets are an emerging biomedical model for cystic fibrosis, lung transplantation, influenza, and diabetes (van Riel et al. 2006; Tumpey et al. 2007; Tripp and Tompkins 2009; Sun et al. 2010; Olivier et al. 2012; Sui et al. 2013; Peng et al. 2014). Intraspecific SCNT in mustelids is now a regular feature of biomedical research which makes iSCNT in the genus *Mustela* worthy of investigation.

## iSCNT in Black-Footed Ferrets

Reproductive cloning research in endangered species has largely relied on iSCNT; the large number of donated oocytes needed and the invasive nature of collection often make it unfeasible to use endangered species as oocyte donors, as is the case with the black-footed ferret. Developing interspecies SCNT protocol with the goal of restoring genetic diversity to the species will require a dedicated effort with a well-planned series of experiments aimed at developing healthy black-footed ferret kits (Figure 2). In particular, experiments aimed at increasing blastocyst development efficiency will decrease the likelihood of downstream developmental abnormalities.

A first consideration is choice of species for donor oocytes. Success of iSCNT will depend in part on the compatibility of donor cytoplasmic material with black-footed ferret nuclear material to develop a full-term cloned animal; therefore, careful consideration of the costs and benefits of each potential donor species will need to be made. The most closely related species to black-footed ferrets are steppe polecats (*Mustela eversmanni*) which diverged approximately 800000 years ago from black-footed ferrets (O’Brien et al. 1989). This species, although common in the wild, would require the additional development of reproductive and animal husbandry resources to breed in captivity while developing iSCNT resources for black-footed ferret. Domestic ferrets, descended from the European polecat, are congener to black-footed ferrets and are regularly used in reproductive cloning, and therefore, the technical aspects of oocyte harvesting and enucleation have already been developed (Li et al. 2006).

It is unknown if epigenetic or genetic (nuclear vs. mitochondrial) incompatibilities of black-footed ferrets with either Steppe polecats or domestic ferrets would inhibit the production of a viable, full-term animal. Indirect evidence suggests that the likelihood of creating a viable interspecies blastocyst is high, given that incomplete reproductive isolation is a feature of this clade. Other mustelids in the ferret clade hybridize in captivity (Amstislavsky and Ternovskaya 2000) and the wild (Cabria et al. 2011). Mustela *lutreola* (2*n* = 38) and *M. putorius* (2*n* = 40) show strong evidence of F1 hybridization and backcrossing in the wild, which suggests that F1 animals are fertile (Cabria et al. 2011). *Mustela nigripes* (2*n* = 38) have previously been bred with domestic ferrets (*M. putorius furo*; 2*n* = 40; Biggins D, personal communication), and *M. nigripes* × *M. eversmanni* (2*n* = 38) crosses produced healthy offspring (Williams et al. 1996; Davison et al. 1999). *Mustela nigripes* and *M. eversmanni* are considered ecological surrogates (Biggins et al. 2011) and *M. nigripes*, *M. eversmanni*, and *M. putorius* have been hypothesized to be one Holarctic species. Given the extensive amount of hybridization that has occurred in nature and in captivity, it appears that epigenetic and genetic compatibility is a feature among mustelids. It should be noted that because nuclear genomes are not admixed in reproductive cloning, chromosomal differences among species should not impact the success of iSCNT.

For the sake of further discussion of iSCNT in ferrets, we will assume the domestic ferret to be the oocyte donor species, with the understanding that other ferret species may ultimately be found to be more suitable for iSCNT. To clone black-footed ferrets, domestic ferret oocytes would need to be harvested, enucleated, and fused with the fibroblast of black-footed ferrets (Figure 1). The resulting hybrid oocyte would be allowed to develop *in vitro* until the blastocyst stage and then implanted into a surrogate mother. Finding the appropriate species (domestic ferret, black-footed ferret, or a hybrid) would require embryo transfers to determine if healthy animals of one species could be carried to term and born to another species. For SCNT cloning of domestic ferrets, the transfer of the embryos to the surrogate requires surgery in order to place the embryos in the oviduct. The risk of surgery may make highly endangered black-footed ferrets a less desirable surrogate mother.

Ultimately, the product of this iSCNT would be animals with black-footed ferret nuclear DNA and a mix of mitochondrial DNA from both donor species. The resulting interspecies clone would be subject to all of the inefficiencies and abnormal development inherent in iSCNT as discussed above. *In vitro* experiments whose goal was to reduce improper genetic reprogramming to increase healthy blastocyst and ultimately fetal and juvenile development would need to be undertaken. As discussed earlier, promising use of treatments to erase methylation patterns seem to mimic the process of genetic reprogramming and hold promise for endangered species iSCNT (Gómez et al. 2012). Experiments that aim to understand the developmental repercussions of heteroplasmic oocytes that have both the mitochondria of the oocyte donor and the nucleus donor would address the potential problems of mitochondrial/nuclear genome compatibility.

In order to produce a black-footed ferret with entirely native cytoplasmic features, male clones, which were the product of iSCNT, would need to be mated with wild-type female black-footed ferrets. All resulting F1 progeny would have the maternally inherited black-footed ferret mtDNA and maternally derived cytoplasm and a recombination of cloned and captive black-footed ferret nuclear DNA which would begin the process of genetic restoration. To capture all of the genetic variability in the cloned male, multiple F1 individuals would need to be produced since each F1 would contain 50% of cloned black-footed ferret DNA.

For cloned females, the process would take one extra generation to produce black-footed ferrets without domestic ferret cytoplasmic features. The cloned females would be mated with black-footed ferret males to produce F1 progeny that all had domestic ferret mitochondrial genomes and cytoplasm. Only F1 males would then be mated to wild-type black-footed ferret females to produce F2 progeny with black-footed ferret mitochondrial genomes and cytoplasm. Multiple F2 progeny would be needed to capture all of the genetic variability of the donor black-footed ferret genome. By these breeding methods, domestic ferret mitochondrial genes would theoretically be purged from the resulting progeny.

Key to the success of any conservation cloning efforts would be proactive decision making and strategic planning about who would oversee the process and how decisions would be made (Figure 2). As with any endangered species, access to black-footed ferret biomaterials or live animals would require proper permitting, and live animals would need to be cared for by qualified institutions that can meet the unique animal husbandry requirements of this species.

Once live animals were born and maintained in captivity, clones and their resulting lineages would need to be carefully monitored for signs of embryonic, developmental, and physiological abnormalities throughout the course of their lifetimes and over multiple generations to assess the intra- and intergenerational legacies of epigenetic, genetic, and maternal effects of cloning. Decisions about whether and when the program was ready to incorporate descendants of cloning back into the captive-breeding program would need to be made in an informed and scientific manner in consultation with the Species Survival Plan. If no observable adverse effects were found, then the recovery program could consider promoting the new genomes throughout the captive and reintroduced populations of black-footed ferrets. Increased genetic diversity and increased fitness would be the hallmark of genetic restoration and rescue from inbreeding depression via iSCNT cloning.

## Source Material for Cloning: Which Material Is the Best Choice?

Cloning black-footed ferrets would only be valuable if genetic material that could replenish the black-footed ferret gene pool were available in a form that could be used in iSCNT. Several sources exist, and the most efficacious of these resources are cryopreserved fibroblast cells. These cell cultures were established and frozen in the San Diego Zoo Institute for Conservation Research’s Frozen Zoo^®^ from 2 black-footed ferrets from the ancestral Meeteetse, Wyoming, population. Skin biopsies were obtained from 1 male and 1 female, and fibroblast cell cultures were established and successfully frozen as demonstrated by cell growth following thawing. Cytogenetic analysis revealed 2*n* = 38 chromosomes (Ryder et al., in preparation). Fibroblasts are regularly used as sources of nuclear material for SCNT and thus have a high possibility of success compared to other sources of somatic material. In addition to their feasibility as source genomic material, these cell lines were collected from individuals that did not directly contribute to the foundation of the captive breeding population, and thus inclusion of their genetic material in the current gene pool would represent an increase to standing genetic diversity in the black-footed ferret gene pool. The relatedness of these 2 cell donors to each other or to the genetic founders of the extant population is unknown, and thus, the magnitude of their potential contribution to genetic diversity is currently unknown. Nonetheless, as has been demonstrated repeatedly in other cases of genetic rescue, the addition of variation from even 1 individual can change the demographic trajectory of a population (e.g. wolves from the Italian Alps; Vila et al. 2003). For conservation captive breeding, animals with low mean kinship that have high genetic uniqueness compared to the rest of the breeding population will always make valuable contributions to the variability of the gene pool. While these additions to the gene pool of black-footed ferrets could alleviate symptoms of inbreeding depression, the likelihood of the addition contributing to outbreeding depression is remote. Because the donated genomes would be from the same population as the founders of the captive population, there would be minimal concern about disrupting coadapted gene complexes or other effects of outbreeding.

A complicating factor for one of these cell lines is that the male black-footed ferret, from which one cell line was established, died from CDV 7 days after being biopsied. Because CDV is an intracellular virus, it is very likely that cell cultures from this individual are also infected with CDV. Because both cell cultures were derived from black-footed ferrets that were exposed to and in one case died from CDV, cell cultures of both lines should be tested for the presence of CDV. CDV is highly pathogenic to ferret species, and its presence would complicate cloning. CDV vaccines have been developed and are regularly used in black-footed ferrets. Antiretroviral therapy could also be used to restrict vertical transmission (Cooper et al. 2002).

Additional sources of genetic material are cryopreserved tissues and semen from deceased animals from early generations of captive breeding. These animals are genetically valuable as they have high genome uniqueness compared to the extant population that is 20 generations older. Early generation individuals would have low mean kinship and high genome uniqueness compared to extant individuals.

While early attempts at cloning should use the highest quality source genetic material possible from cryopreserved material, future endeavors may be able to use tissue from other sources. These tissues include skeletal tissue that has been frozen but not cryopreserved from the extinct Mellette County, South Dakota, population. This population is estimated to have been isolated from the Meeteetse (founding) population for 5000–8000 years as intermontane grasslands became separated from Great Plains grasslands by high elevation sagebrush habitat (Wisely et al. 2008). The genetic material from South Dakota would represent a unique genetic contribution, but the effects of outbreeding would need to be considered. Local adaptation to the environment could have led the peripheral Meeteetse population down a unique evolutionary pathway that differentiated it from the Great Plains populations; rapid evolutionary change has been suggested for another North American mustelid, the Pacific fisher (*Martes pennanti*, Wisely et al. 2004). A fuller understanding of genomic differentiation among these populations and the consequences of introducing genetic material from one population to another is needed.

New genomic resources that may eventually benefit conservation cloning have been discussed in scientific forums (e.g. Friese 2013; Fletcher 2014; Jones 2014) but have yet to be successfully practiced. Reconstruction of ancient genomes from low-yield, low-quality DNA sources such as museum specimens, permafrost, and sub-fossil material has become routine (Fletcher 2014). Using contemporary genomes as a scaffold, it is possible to assemble whole extinct genomes from ancient sources. The technological gap exists at the juncture of turning template DNA into chromatin that would be injected into an enucleated oocyte. Whole genomes would need to be assembled with histones into supercoils of chromatin that are assembled into chromosomes, and that technology is not present at this time. 

For black-footed ferrets, short fragments of mtDNA from museum specimens have been successfully extracted (Wisely et al. 2004) and utilized for phylogeographic reconstruction (Wisely et al. 2008), and hundreds of museum specimens exist for whole genome reconstruction of extinct genomes of black-footed ferrets. Four black-footed ferret genomes have been sequenced (http://www.ncbi.nlm.nih.gov/sra/?term=SRP044096) and could be used as a scaffold to understand and reconstruct genomic variation across the geographic breadth of this species. These endeavors unto themselves will be valuable to understanding the nature of loss of genetic variation.

## Costs and Benefits of Gene Pool Enrichment via iSCNT

With the introduction of any source of new genetic variation in *ex situ* populations of endangered species, positive or negative fitness consequences may occur. Gene pool enrichment has been observed to increase population viability through increased fecundity, juvenile survival, recruitment, and immunocompetence in both facilitated translocation and natural migration events (Vila et al. 2003; Pimm 2006; Heber 2013). Genetic analysis of translocation and migration events has documented changes in genetic diversity and demography, even from the contribution of a single individual (Adams 2011; Vila et al. 2003). While benefits have been observed and the process by which these benefits arise is modeled, the role of evolutionarily significant genes in these events is still not well characterized, as most studies have focused on neutral genetic variation for analysis (Steiner et al. 2013; Vander Wal et al. 2013). Understanding the underlying mechanisms of how introduced alleles improve population quality is of increasing interest to conservation and requires the ability to link genotype, phenotype, and demography (Vander Wal et al. 2013).

The benefits of gene pool enrichment to the black-footed ferret include the potential to increase fitness through the decrease of inbreeding, the accompanying reduction in homozygosity across the genome, and an increased adaptability by restoring lost genetic variation. Developing these techniques in advance of a catastrophic population decline or to thwart the slow, inevitable erosion of genetic diversity in the captive-breeding population offers means to enhance the future prospects of this species that would not otherwise be available (Piña-Aguilar et al. 2009). Successful development of reproductive cloning is the only path to providing an influx of unrepresented genomes into the extant lineage of black-footed ferrets.

Genetic enrichment or genetic rescue is likely to be called upon for recovery of other endangered species. The experiences gained in developing and validating the application of advanced genetic and reproductive technologies in the black-footed ferret will be illustrative and potentially serve as a model for other species. Although the potential for contributing to species recovery is apparent but unproven, prospects of a continuing decline in fitness and risk of catastrophic events such as disease outbreaks urge initiation of the evaluation of the cell cultures, frozen tissues, and museum specimens as recovery tools for the species.

The risks associated with advanced genetic and reproductive technologies to sustain black-footed ferret recovery efforts include the possibility of introducing nonadaptive or deleterious phenotypes into the population. These maladaptations could be caused by classical inheritance due to disruption of co-adapted gene complexes or non-genetic but heritable causes such as epistatic effects or genome incompatibility. The evaluation of genetic material for genetic enrichment or rescue is largely unexplored territory, although it is experimentally approachable in related species and model organisms. An additional consideration is the possibility of the introduction of intracellular viral diseases. A thorough understanding of the implications of a cell line infected with CDV is needed. Screening of any biological material used for SCNT for potential pathogens is warranted and would be an essential step in the process of decision making.

Critics of using reproductive cloning in endangered species management claim that the cost of the technology will divert conservation resources from more holistic and fruitful efforts of conserving habitats and keeping common species common (Ehrenfeld 2006). The cost of developing a reproductive cloning program would include programs to 1) systematically evaluate the efficiency and feasibility of interspecies blastocyst production, 2) evaluate techniques that may increase blastocyst survival, including experiments to avoid improper reprogramming, or the efficacy of making heteroplasmic embryos, 3) develop and evaluate the process of producing viable fetuses, and post-parturition animals, 4) house, breed, and evaluate the health of cloned individuals and their descendants. One promising avenue of funding is the field of biomedicine. Within biomedicine, comparative genome analyses has increased interest in understanding genomic variation and gene function in model species’ congeneric relatives (Carneiro et al. 2011; Peng et al. 2014). More directly, interest in iSCNT as a tool for understanding embryology and early development promise advancements in understanding genome incompatibilities and epigenetic effects of iSCNT (Sun and Zhu 2014), which is currently a major impediment to the production of healthy iSCNT clones.

## Recommendations

Endangered species recovery involves many stakeholders with differing perspectives on which aspects of recovery should be emphasized, and genetic restoration as a recovery tool can be controversial even without hot button issues like conservation cloning. If genetic restoration is being considered, we recommend a formal structured decision-making process to allow stakeholders to define specific problems inhibiting recovery, identify objectives to move recovery forward, and evaluate the possible solutions prior to implementing them (Wilson and Arvai 2011). Decisions based on knowledge of the state of the science, and the risks, benefits, and trade-offs have been shown to increase the success of outcomes and the satisfaction of stakeholders. This process will help stakeholders determine the necessity of genetic restoration and if reproductive cloning is a worthwhile tool that provides a solution to the defined recovery problem.

Should reproductive cloning be found to be a conservation-worthy endeavor, we recommend that a formal logical framework (Cordingley 1995) be used to guide the process. Logic frameworks are used to clearly define and then integrate numerous, complex tasks associated with well-defined objectives. Short-, medium-, and long-term objectives are articulated, activities are constructed, and resources are identified to achieve the defined goals (Julian 1997). From the logic framework, stakeholders will obtain defined steps on the roadmap of genetic rescue (Figure 2).

For black-footed ferrets, the causes of maladaptive phenotypes found in the captive population are not defined. Environmental, genetic, or some combination of these 2 phenotypic drivers could be acting to decrease fitness in the captive population. Even without causative evidence of inbreeding depression, an infusion of genetically unique individuals would increase diversity in the gene pool, lengthen generation time, and decrease inbreeding. The captive-breeding program will need to continue producing individuals for the foreseeable future, and genetic restoration would be one step toward maintaining a viable captive-breeding population and a wild population that is resilient to future environmental changes. The authors recommend a formal structured decision-making framework (Martin et al. 2009) that includes all stakeholders to understand and define the problems in the captive black-footed ferret population, and weigh the usefulness, benefits, hazards, and costs of incorporating genetic restoration including iSCNT into the recovery plan of the black-footed ferret.

## Significance to Conservation

Significant public and private resources have been devoted to black-footed ferret recovery. Against the odds of extinction by disease, genetic erosion, and poor founder reproduction, many citizens, scientists, veterinarians, zoos, wildlife managers, wildlife agencies, and other governmental bodies have worked together to bring the black-footed ferret back from the brink of extinction. Appropriately iconic, this species serves as an example of how anthropogenic pressures can decimate species, but also how perseverance and community cooperation can rescue these rare species from extinction. Black-footed ferret recovery has led the way in promoting advanced assisted breeding in the conservation and recovery of critically endangered wildlife.

Reproductive cloning holds promise as a tool for conservation. If practitioners responsible for these efforts proceed using science, sound decision making, and outcome oriented logic models to move forward, then the entire conservation community can benefit. Multiple canid, felid, and ungulate species have well-studied reproductive biology with well-developed assisted breeding technologies and make good candidates for reproductive cloning (reviewed in Piña-Aguilar et al. 2009). We hope that this article provides a way forward for sound decision making in incorporating advance reproductive science techniques into conservation breeding.

## Funding

University of Florida’s Department of Wildlife Ecology and Conservation, Lincoln Park Zoo, San Diego Zoo Institute for Conservation Research, and supporters of Revive and Restore (http://longnow.org/revive/our-supporters/).
